# Surgery 4.0: From the Smart Operating Room to the Learning Operating Room

**DOI:** 10.3390/jcm15166384

**Published:** 2026-08-18

**Authors:** Andrew A. Gumbs, Roland Croner, Jean-Claude Couffinhal

**Affiliations:** 1 Department of General, Visceral, Vascular and Transplant Surgery, University Hospital Magdeburg, 39120 Magdeburg, Germany; 2 Service de Chirurgie Digestive Minimale Invasive, Hôpital Antoine Béclère, Assistance Publique-Hôpitaux de Paris, 157 Rue de la Porte de Trivaux, 92140 Clamart, France; 3 Commission Innovation, Académie Nationale de Chirurgie, Les Cordeliers, 15 Rue de l’École de Médecine, 75006 Paris, France; 4 Do Tank Chirurgie 4.0—“La Fabrique de la Chirurgie Numérique”, 75017 Paris, France

**Keywords:** Surgery 4.0, learning operating room, surgical world model, artificial intelligence in surgery, surgical autonomy, cognitive governance, digital health sovereignty, federated learning

## Abstract

The connected operating room captures, transmits, and displays data, but it does not learn. This perspective presents Chirurgie 4.0 (C40), a French-led initiative born within the Commission Innovation of the Académie nationale de chirurgie, which proposes not only a concept but a method for the safe adoption of artificial intelligence (AI) in surgery. At its core is the C40 Maturity Model of the Operating Room, a human-governed “surgical world model” describing the transition from the Smart OR to the Learning OR across six levels, from the conventional operating room to a sovereign, federated network of surgical world models. We situate surgical autonomy on an explicit six-level scale, show that autonomous devices are already an accepted clinical reality in fields such as interventional cardiology, ophthalmology, neuro- and orthopedic surgery, and argue that governance must be native rather than retrofitted, through a Cognitive Governance Layer resting on human oversight, explainability, auditability and agent governance. We describe the economic and sovereignty stakes specific to intelligent surgical technologies and set out the design of the 2026 C40 field survey, whose results will feed a Livre Blanc for public decision-makers. C40 offers five steps that can genuinely be climbed, and a method for climbing them safely, with the surgeon retaining final clinical authority at every step.

## 1. Introduction

There is a recurring danger in surgical innovation: that technology will outpace our capacity to govern it. It has been argued that the European Union urgently needs guidelines for the safe implementation of artificial intelligence (AI) in surgery, and that the obstacles are structural rather than technical. Specifically, surgical data are multimodal and irreducibly non-siloed, “surgomics” resists the clean quantification that has allowed radiomics and pathomics to flourish, and the Medical Device Regulation (MDR) was written for devices that do not change [1,2]. Furthermore, what no editorial has yet supplied is a method for how to evaluate and regulate such dynamic devices in medicine.

A great deal of recent commentary establishes, persuasively, that the current framework is inadequate for software that learns [1,3,4]; far less has been said about how a surgical community should move, and in what order, from the operating rooms it has today to the ones this software will require. The value of a maturity model is that it converts a general anxiety about ungoverned autonomy into a sequence of defined, assessable states, each with its own prerequisites, its own risks, and its own governance obligations.

**Figure 1:**
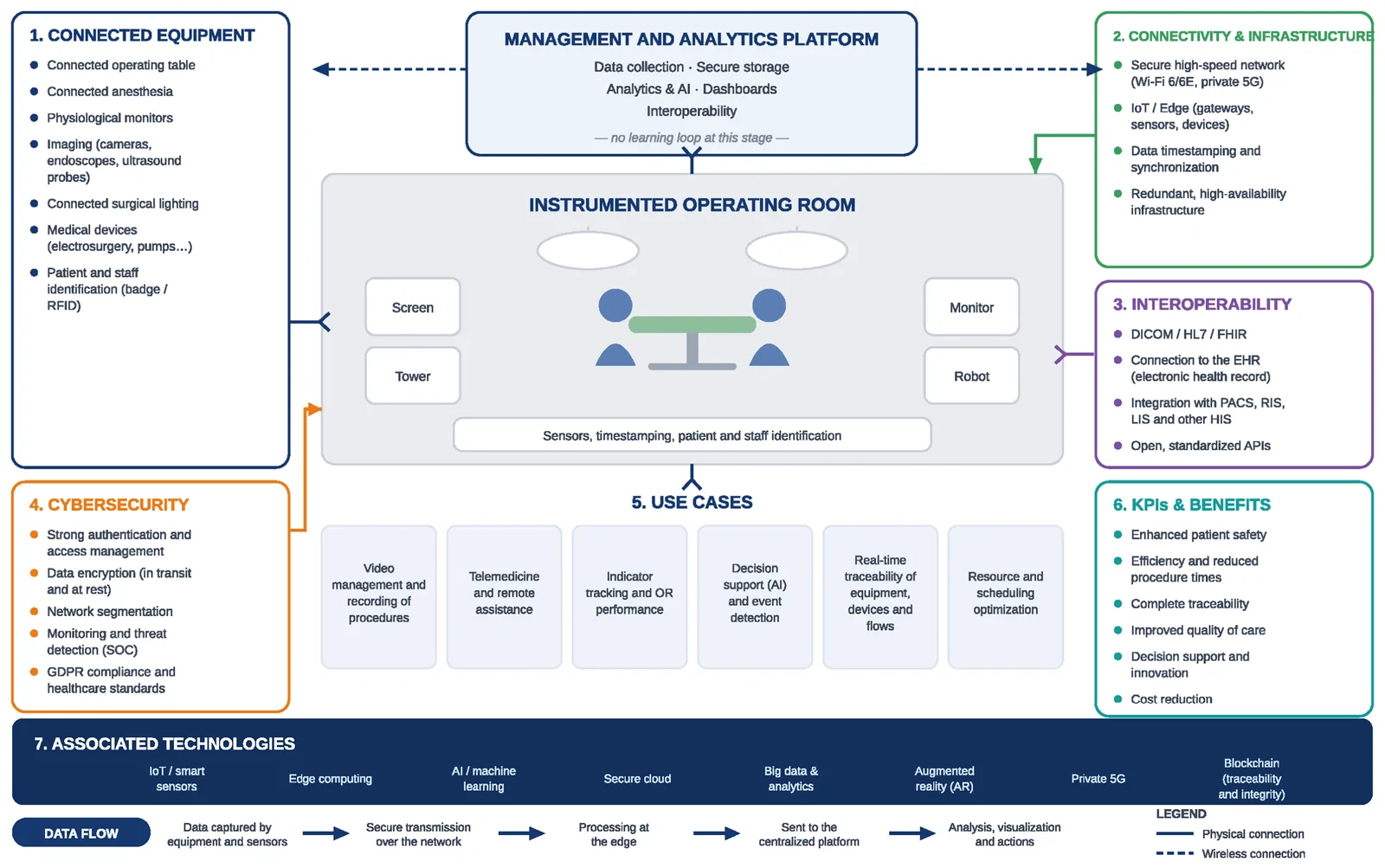
The connected operating room: toward a consensus, interoperable architecture. Chirurgie 4.0 concept. Depiction of the target architecture of a connected operating room, corresponding to levels 2–3 of the C40 maturity model. Seven groupings are represented: (1) connected equipment (operating table, anesthesia, physiological monitors, imaging, lighting, medical devices, patient and staff identification by badge or RFID); (2) connectivity and infrastructure (secure high-speed network, Wi-Fi 6/6E and private 5G, IoT and edge gateways, timestamping and synchronization, redundant infrastructure); (3) interoperability (DICOM/HL7/FHIR, service-oriented device connectivity per IEEE 11073 SDC and ISO/IEEE 11073-10207/-20701, connection to the electronic health record, integration with PACS/RIS/LIS and other hospital information systems, open APIs); (4) cybersecurity (strong authentication, encryption in transit and at rest, network segmentation, monitoring and threat detection, GDPR compliance); (5) use cases (video management, telemedicine, performance-indicator tracking, decision support and event detection, real-time traceability, resource optimization); (6) performance indicators and expected benefits; (7) associated technologies. The whole converges on a management and analytics platform providing collection, secure storage, analytics, and interoperability. This operating room captures and transmits data, but does not yet include a learning loop. Conceived by the Do Tank Chirurgie 4.0; figure rendered with AI assistance. (c) Chirurgie 4.0, reproduced with permission.

**Figure 2:**
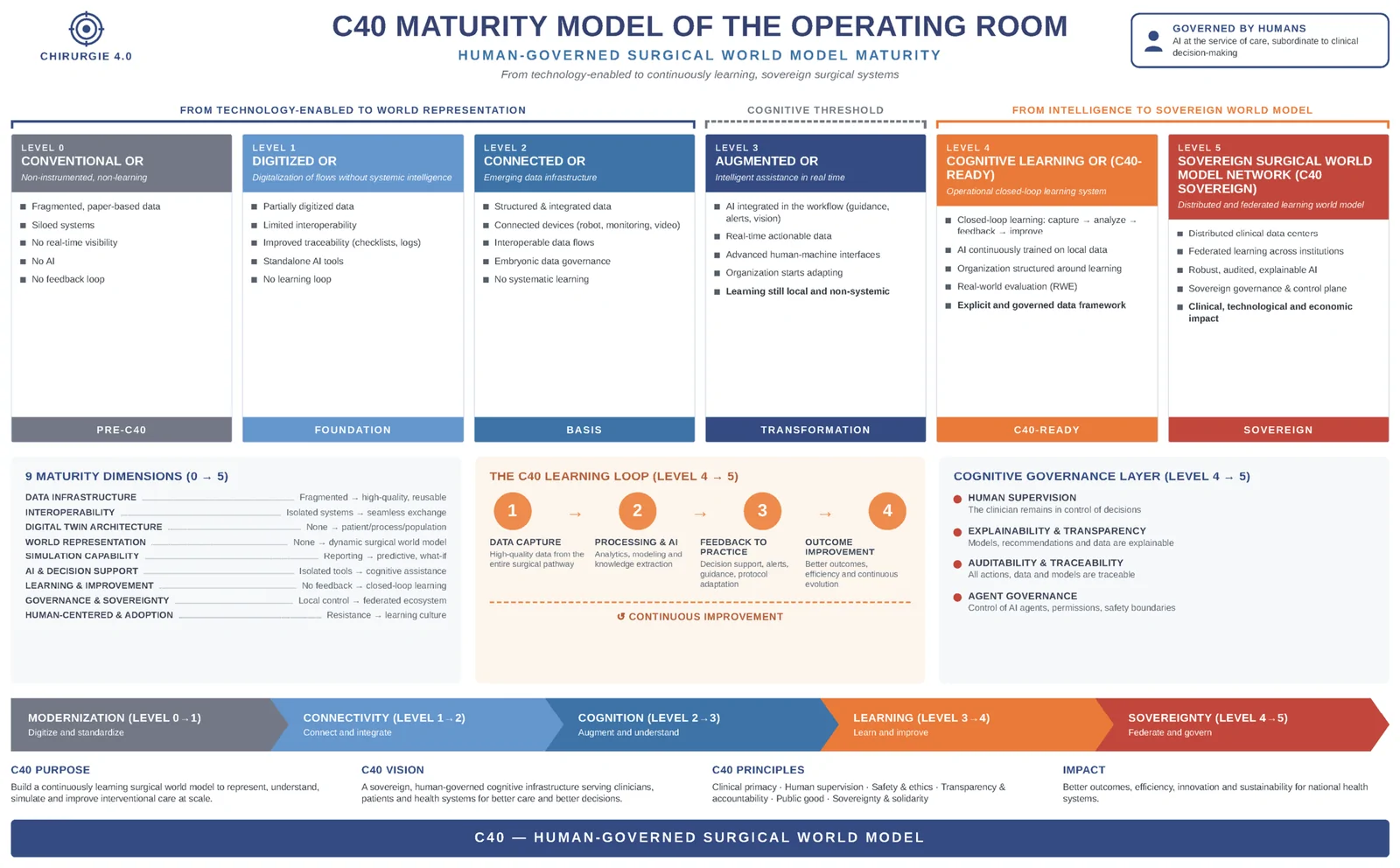
The C40 Maturity Model of the Operating Room: maturity of the human-governed surgical world model. Chirurgie 4.0 concept. A six-level scale (0 to 5) describing the transition from an operating room equipped with technology to a learning, sovereign surgical system. Level 0, conventional operating room (non-instrumented, non-learning); level 1, digitized operating room (digitization of workflows, without systemic intelligence); level 2, connected operating room (emerging data infrastructure); level 3, augmented operating room (real-time intelligent assistance), which constitutes the cognitive threshold; level 4, learning cognitive operating room, “C40-ready” (operational closed-loop learning system); level 5, sovereign network of surgical world models, distributed and federated (“C40 Sovereign”). The five corresponding transitions are modernization (0→1), connectivity (1→2), cognition (2→3), learning (3→4), and sovereignty (4→5). The model is described along nine maturity dimensions: data infrastructure, interoperability, digital-twin architecture, world-representation capability, simulation capability, AI and decision support, learning and improvement, governance and sovereignty, and culture and adoption. From level 4 onward, a Cognitive Governance Layer is activated: human oversight, explainability and transparency, auditability and traceability, and governance of AI agents. Guiding principle: the operating room is governed by humans, with AI serving care and subordinate to clinical decision-making. Conceived by the Do Tank Chirurgie 4.0; figure rendered with AI assistance. (c) Chirurgie 4.0, reproduced with permission.

**Figure 3:**
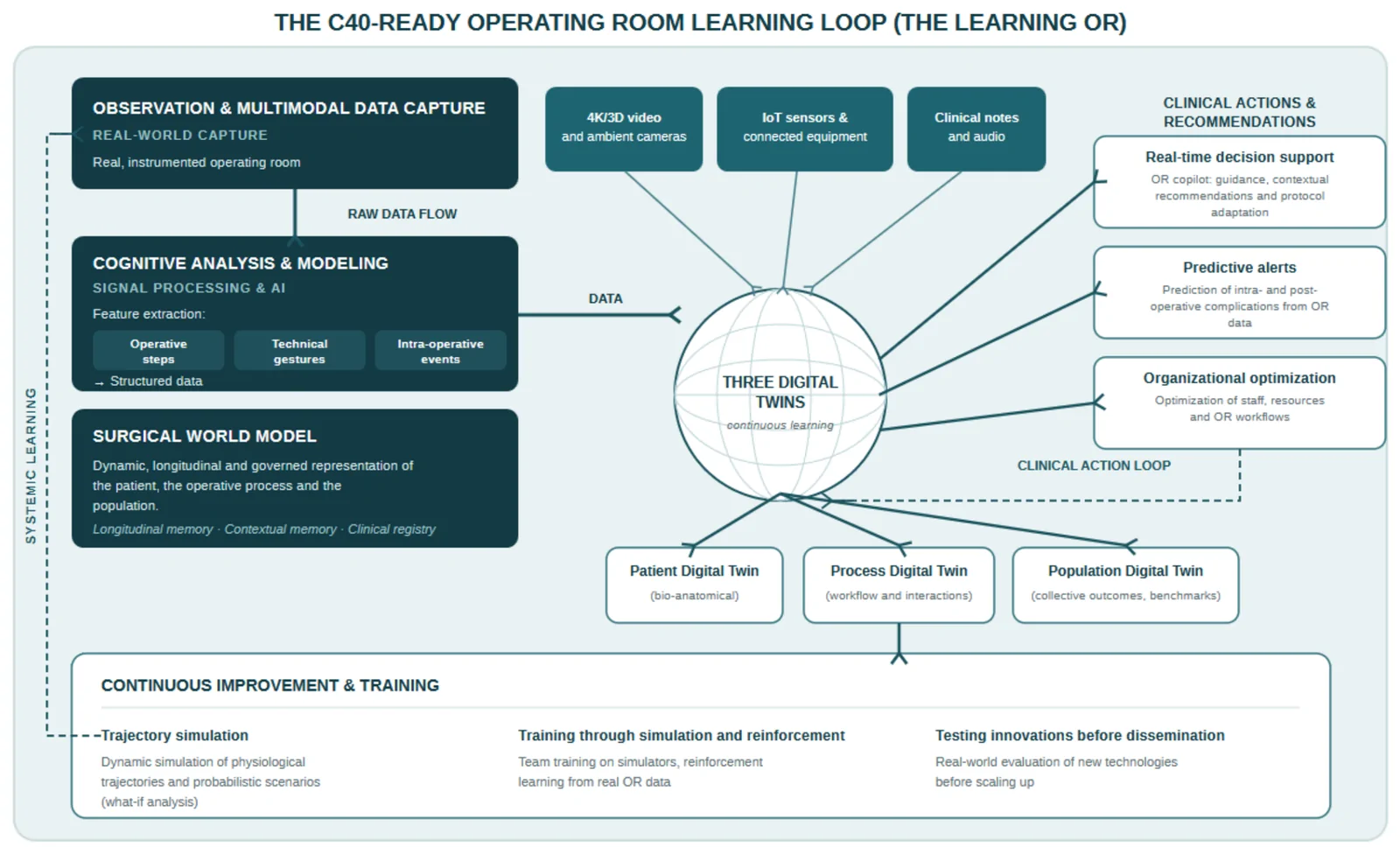
The C40 learning loop of the C40-ready operating room (the learning OR). Chirurgie 4.0 concept. Functional architecture of level 4 of the C40 model. Observation and multimodal capture of the real world (4K/3D video, ambient cameras, IoT sensors and connected equipment, clinical notes and audio) feed a phase of analysis and cognitive modeling (signal processing and AI; feature extraction covering operative steps, technical gestures, and intraoperative events). Before any multimodal stream enters the learning loop, faces, voices, and direct identifiers in the video, audio, and physiological signals are removed or pseudonymized at the edge—on the capture device or local gateway, on-site—so that only de-identified features, and never raw identifiable recordings, are retained or shared across institutions. The structured data feed the surgical world model, organized around three complementary digital twins: the patient twin (bio-anatomical), the operative-process twin (workflow and interactions), and the population twin (collective outcomes and benchmarks), linked by a longitudinal memory and a clinical registry. The clinical action loop delivers, in real time, decision support, predictive alerts for complications, and organizational optimization. A systemic learning loop feeds back into trajectory simulation, training through simulation and reinforcement, and the testing of innovations before dissemination. Conceived by the Do Tank Chirurgie 4.0; figure rendered with AI assistance. (c) Chirurgie 4.0, reproduced with permission.

Chirurgie 4.0 (C40) was conceived as a collective, French-led response to precisely this gap: a concept that describes what the operating room must become, and a method by which surgery can safely adopt it. Born within the Commission Innovation of the Académie nationale de chirurgie, it was established as an association in the autumn of 2025, with the ambition to act as a “Do” Tank in the service of France’s and Europe’s clinical, technological, and economic sovereignty. The concepts presented here—the C40 Maturity Model of the Operating Room, the surgical world model, and the Cognitive Governance Layer—together with Figure 1, Figure 2 and Figure 3, were developed by the Chirurgie 4.0 working group and are its intellectual property; they are presented here with its permission.

This perspective sets out the C40 maturity model, locates it within the questions of surgical autonomy, native governance, and economic sovereignty it necessarily touches, and describes the 2026 C40 field survey by which the working group intends to test its central assumption: that most operating rooms today sit far lower on the maturity ladder than the discourse on “AI surgery” would suggest.

## 2. The Connected Operating Room Is Not Enough

The architecture of a fully connected operating room is by now well described (Figure 1): instrumented equipment, a secure high-speed network, IoT (Internet of Things) and edge computing, DICOM (Digital Imaging and Communications in Medicine)/HL7 (Health Level 7)/FHIR (Fast Healthcare Interoperability Resources) interoperability, connection to the electronic health record, cybersecurity, and GDPR (General Data Protection Regulation) compliance, all converging on a management and analytics platform. Emerging service-oriented device-interoperability standards—notably the IEEE 11073 SDC (Service-oriented Device Connectivity) family, comprising the ISO/IEEE 11073-10207 domain information and service model and the ISO/IEEE 11073-20701 service-oriented medical device architecture—are designed to let heterogeneous operating-room equipment interoperate safely and in real time at the point of care, and form the natural technical substrate for the connected operating room described here. Such an operating room captures, transmits, and displays. But capturing is not learning. Without a feedback loop back into practice, without longitudinal memory, and without explicit model governance, it produces data without producing knowledge: each procedure is recorded and then forgotten, its lessons neither extracted nor returned to the surgeons who might act on them. The missing element is not more sensors but a governed loop, under explicit human control and with an auditable memory.

Europe has shown that the missing layer can be built. EUCAIM (EUropean Federation for CAncer IMaging), the flagship federated imaging infrastructure of the European Cancer Imaging Initiative, demonstrates that hospitals can retain their data locally while sharing derived features at scale [5]. Nothing of the kind yet exists for the operating room, whose data are more heterogeneous, more temporally structured, and harder to de-identify than a stack of images. This is the limit that Chirurgie 4.0 seeks to cross, and the reason it insists that the operating room, not the algorithm, is the correct unit of analysis.

## 3. The Learning Operating Room, Step by Step

At the heart of the concept lies the C40 Maturity Model of the Operating Room (Figure 2), a human-governed “surgical world model” that turns the ambition of an intelligent operating room into a ladder that can be climbed. Its principle is unambiguous: the operating room is governed by humans, with AI serving care and subordinate to clinical decision-making. Its motto sums up the shift: we move from the Smart OR to the Learning OR. Crucially, it presents the transformation in five transitions rather than as one impossible leap.

The first stage is modernization: moving from the conventional operating room, non-instrumented, non-learning, with fragmented paper data and siloed systems, to a digitized operating room where workflows are captured and traceability improves. The second is connectivity: the connected operating room, where data become structured and interoperable, robots and monitors are networked, and data governance emerges. The third is cognition, the true threshold: the augmented operating room, where AI is integrated into the workflow through guidance, alerts, and computer vision, and the organization begins to adapt around it [6]. The fourth is learning: the “C40-ready” cognitive operating room, the first stage at which the operating room genuinely closes the loop, from capture to analysis to feedback into practice to improved outcomes, trained on local data, with real-world evaluation and an explicit, governed data framework. The fifth is sovereignty: a distributed network of surgical world models, with federated learning across institutions and audited (explainable AI).

The model is deliberately multidimensional, describing progress along nine maturity dimensions rather than a single axis of “how much technology is present” because operating rooms mature unevenly: an institution may possess sophisticated imaging integration yet have no learning loop, or strong data governance yet weak clinical adoption. Level 4 is detailed in the C40 learning loop (Figure 3), whose architecture rests on three complementary digital twins: the patient (bio-anatomical), the operative process (workflow and interactions), and the population (collective outcomes and benchmarks). Their interaction enables continuous learning and delivers concrete clinical value: predictive alerts for intraoperative complications, decision support in real time, organizational optimization, and a longitudinal memory that lets a team compare its practice against its own past and against its peers.

The converging impression of the working group is that the great majority of French operating rooms today sit at levels 1 and 2, with only a few institutions reaching levels 3 to 4; most are internally connected, but without the federated interconnection that would allow medical knowledge to be produced and shared between institutions. This impression needs to be documented, one aim of the survey described below. The extension of the model from the operating room to the surgical system as a whole, along the dimensions of clinical impact, technological sovereignty, and public health strategy, is the subject of separate work by the Chirurgie 4.0 group.

## 4. Where Autonomy Actually Sits

One of the reasons clarity matters so much is that the vocabulary of “robotic surgery” is overloaded with meaning [7]. Most of what is called robotic surgery is in practice tele-manipulation: level 1 on the six-level scale of surgical autonomy (not to be confused with the C40 maturity levels), with the operation entirely under the surgeon’s control [1,8]. Levels 2 to 4 introduce increasing degrees of autonomous action under surgeon supervision; only level 5 denotes complete autonomy, with no surgeon (human) in the loop [8].

Neither of the higher autonomy levels is hypothetical. Laser refractive surgery already operates at level 4: once the surgeon has set the parameters and docked the eye, the femtosecond and excimer lasers execute the flap and the stromal ablation as a programmed sequence under real-time supervision, not by hand. The same pattern recurs across specialties. In orthopedic surgery, active robotic systems have for years performed autonomous milling of bone: once the surgeon validates a preoperative plan and registers the anatomy, the robot removes bone to prepare the implant cavity as a programmed sequence, under supervision but not by hand, again a level-4 action on tissue. In neurosurgery, stereotactic radiosurgery delivers a preplanned ablative dose to an intracranial target autonomously, with the robotic platform executing the tissue-modifying step while the clinician supervises rather than performs it. Even if such autonomy is programmed rather than learned, in each case it is the machine and not the hand that carries out the tissue-modifying steps. The cardiac pacemaker and AICD (Automatic Implantable Cardioverter-Defibrillator) function at level 5 in all but name: they sense, decide, and act on the patient continuously, for years, with no clinician in the loop. Autonomous devices are therefore not a frontier we are approaching but a reality we have already accepted—in ophthalmology, cardiology, orthopedics, and neurosurgery—without ever naming it as such. The real task is not to prohibit autonomy but to decide, deliberately and transparently, where each surgical technology sits on this scale and how that level is validated [9].

The operating room is safe only when the level of autonomy of every tool within it is named, evaluated, and governed. The C40 maturity scale provides that framework, and imposes a shift in perspective: the robot is not the center of the operating room but one instrumented node among others, and autonomy is not a marketing argument but a governed property—declared, evaluated, supervised, revisable.

## 5. Governance Built in, Not Bolted on

The governance of such a system cannot be a late addition. The MDR assumes a device that remains stable once certified, whereas a learning system may drift, retrain itself, or silently degrade; it cannot govern software that changes after approval [1]. A maturity model that explicitly names the “learning” and “sovereign” stages, and wraps them in a governance layer, is better suited to evolving software than any one-off certification, and it supplies operational scaffolding for the call to action proposed in the British Journal of Surgery [1]: auditable explainability, procedure-specific validation, unified data standards, adaptive monitoring with automatic suspension thresholds, and responsibility shared among surgeons, institutions, and manufacturers. Independent work reaches the same conclusion from a different direction [3].

This is what distinguishes the concept from mere technological optimism: governance is native to it. At the learning and sovereignty stages, the model activates a Cognitive Governance Layer resting on four pillars (Figure 2): human oversight, whereby the clinician retains control of decisions; explainability and transparency, so that models, recommendations, and data are comprehensible; auditability and traceability, so that every action, dataset, and model can be tracked; and agent governance, which sets the permissions, safety limits, and escalation rules for AI agents. To this must be added the cybersecurity of the operating room itself (Figure 1): a learning operating room is, by construction, an exposed one, and its security is a condition of its clinical legitimacy, not an integration option.

Two recent contributions reinforce this orientation: a JAMA perspective arguing that autonomous clinical AI should be licensed and continuously reassessed rather than approved once as a device [4], and a reflection in the Journal of Robotic Surgery insisting that judgment and responsibility remain irreducibly human [10].

## 6. The Economic Break and the Sovereignty Stake

A learning operating room will only come into being if the innovations that equip it actually reach the patient, and this is precisely where the French system seizes up. Seed funding is secured, notably through France 2030, but a break then appears, for lack of evaluation, reimbursement, and market-access pathways suited to intelligent surgical technologies. The result: French gems, developed with public funds, raise foreign capital and are acquired abroad, risking their datasets and know-how falling under extraterritorial legislation. This dependence is as much a matter of health sovereignty as of industrial policy. Beyond financing, two practical barriers that the maturity model makes explicit must also be crossed: the up-front infrastructure investment required to climb from levels 1–2 to the connected and augmented operating room, and the workforce training and change management without which even well-equipped teams cannot close the learning loop.

Organized into task forces around five building blocks and bringing together some 70 experts from across the ecosystem, Chirurgie 4.0 puts forward five concrete responses, submitted for political arbitration: a national observatory of surgical and interventional innovations, to provide a quantified map of the sector; a national early-access pathway for digital, robotic, and algorithmic innovations, with provisional nomenclature and tariff and a three-year experimental registry; a move from centralized to distributed evaluation in accredited centers; recognition and funding of clinical registries as strategic national infrastructure, integrated into the PMSI (Programme de médicalisation des systèmes d’information); and, most structuring of all, the evolution of the Haute Autorité de santé (HAS) into an “augmented HAS” for devices that learn. These measures determine France’s ability to retain mastery over its surgical data.

The technical foundation already partly exists. The TRUST4CURE project, selected as an Important Project of Common European Interest (IPCEI), is building a sovereign health data infrastructure—precisely the environment the learning operating room requires—and the consortium has expressed the wish to integrate Chirurgie 4.0 as its first demonstrator, with prototypes envisaged at the IHU (Institut hospitalo-universitaire) de Strasbourg and in cardiac surgery at AP-HP, Pitié-Salpêtrière. If confirmed, the C40 vision will have a full-scale clinical validation site, and we will move from concept to proof.

## 7. Materials and Methods: The 2026 Chirurgie 4.0 Survey

The 2026 C40 survey is a prospective, mixed-methods field study combining a structured mapping of intelligent surgical technologies with a qualitative and quantitative survey of the surgical ecosystem, conducted by the “Régulation et Évaluation” working group. Its scope comprises the intelligent health technologies (technologies de santé intelligentes, TSI) that are CE-marked, undergoing marking, or whose certification is expected within a 24-month horizon. The survey addresses three questions: whether there is an organizational impact specific to these technologies; what their economic model might be; and whether sector-specific guidelines are needed for the implementation of the European AI Act.

Data are collected through a questionnaire in four parts: a TSI identification form based on the joint guide of the HAS and the Commission nationale de l’informatique et des libertés (CNIL); AI Act implementation and cybersecurity; organizational impact; and economic model. Respondents include surgeons and operating room teams, manufacturers, hospital management, tiers-lieux d’expérimentation, IHU and expert centers, health economists, Conseils nationaux professionnels, and patient associations. Recruitment is open and community-based rather than probabilistic; the working group aims to enroll at least 150 respondents overall, with a minimum of approximately 30 per principal stakeholder category, a target judged sufficient to characterize organizational and economic patterns while remaining feasible within the field-collection window. Because recruitment is non-probabilistic, the representativeness of the sample is itself reported as a study outcome.

Field data collection runs from August to September 2026 through interviews and the structured questionnaire; the working groups process the data from October to November 2026. Quantitative responses will be summarized with descriptive statistics stratified by respondent category and, where possible, by the C40 maturity level of the reporting institution; qualitative material will be analyzed thematically, with coding supported by a large language model (Claude, Anthropic) and independently reviewed and validated by the working group. Any use of AI tools in the analysis will be disclosed in accordance with journal policy. Informed consent will be obtained from every respondent prior to participation, following a participant information notice describing the purpose of the survey, the data collected, their intended use, and the right to withdraw; personal data are processed in compliance with the GDPR. The results will feed the Livre Blanc on the digital transformation of surgery, planned for presentation to public decision-makers in December 2026, followed by an evaluation phase in 2027.

## 8. Discussion

The C40 survey does not stand alone. It extends earlier specialty surveys, such as the E-AHPBA study on the ethics and trustworthiness of AI in hepato-pancreato-biliary surgery [11], and runs in parallel with European initiatives such as the COMPASS-AI project, supported by the EU4Health Programme 2021–2027, whose purpose is to develop guidelines for the safe implementation of AI in healthcare [1]. Like Chirurgie 4.0, COMPASS-AI rests on a multidisciplinary community, seeks to derive good practice from the field and from evidence rather than imposing it from above, and places patient safety and human oversight at its center. The difference is scale: COMPASS-AI is European and covers healthcare as a whole, whereas Chirurgie 4.0 is anchored in the French surgical ecosystem and can be seen as its sectoral, national counterpart.

Three features distinguish the C40 contribution. Its unit of analysis is the operating room rather than the algorithm, making it directly actionable by surgeons and hospital directors, not only regulators. Whereas established healthcare-digitalization maturity models such as the HIMSS (Healthcare Information and Management Systems Society) Electronic Medical Record Adoption Model (EMRAM) and its infrastructure and analytics counterparts assess the hospital or enterprise as a whole, the C40 model takes the operating room as its unit of analysis and adds explicit learning loop and governance dimensions that those general frameworks do not capture. It treats autonomy and governance as properties to be declared and validated at defined maturity levels rather than as binary states to be permitted or forbidden. It also couples concept to method, pairing the maturity model with an empirical instrument to measure where the ecosystem stands and a demonstrator to test the model clinically. The principal limitation is the same one the work is designed to remedy: the claim that most operating rooms sit at levels 1–2 is at this stage an expert impression, not a measured fact, for which the 2026 survey exists to convert into evidence. The model itself will require external validation, both of inter-rater agreement and predictive validity.

## 9. Future Directions

Three lines of work follow directly. The first is empirical: completion of the 2026 survey, publication of a documented maturity map of French operating rooms, and the 2027 evaluation phase, which should move the maturity model from a descriptive scale toward a validated assessment instrument. The second is architectural: realization of the TRUST4CURE demonstrator, the first full-scale test of a level-4, C40-ready operating room under real clinical conditions. The third is institutional: the proposed “augmented HAS” and national early-access pathway, without which even a technically mature learning operating room cannot be reimbursed or sustained. Beyond the operating room, the same logic extends to the surgical system as a whole and, ultimately, to the level-5 vision of a federated network of surgical world models. Whether such a network narrows rather than widens global inequalities in surgical care [12] will depend less on the algorithms than on the governance around them.

## 10. Conclusions

The value of this undertaking will depend on the representativeness of the sample, and we therefore call on the whole ecosystem to contribute directly, by September 2026; the “Régulation et Évaluation” working group is open to all. Chirurgie 4.0 proposes five steps that can genuinely be climbed and a method for deciding, on the basis of evidence, how to climb them safely, with the surgeon retaining final clinical authority at every rung. It is one of the most serious collective attempts to date to make the safe adoption of AI in surgery concrete, and it deserves the engagement of the whole community in the months ahead.

On behalf of the Chirurgie 4.0 working group.

## Data Availability

The datasets generated by the 2026 Chirurgie 4.0 survey will be available from the corresponding author upon reasonable request, subject to applicable data-protection and confidentiality provisions.

## References

[1] Gumbs A.A. (2026). Why the European Union urgently needs guidelines for the safe implementation of artificial intelligence in surgery. Br. J. Surg..

[2] Gumbs A.A., Croner R., Abu-Hilal M., Bannone E., Ishizawa T., Spolverato G., Frigerio I., Siriwardena A., Messaoudi N. (2023). Surgomics and the artificial intelligence, radiomics, genomics, oncopathomics and surgomics (AiRGOS) project. Artif. Intell. Surg..

[3] Manzano Rodriguez A., Snoek C.G.M., Schijven M.P. (2025). Bridging the gap: Exposing the hidden challenges towards adoption of artificial intelligence in surgery. Br. J. Surg..

[4] Bergman A., Wachter R.M., Emanuel E.J. (2026). A licensure framework for autonomous clinical AI. JAMA.

[5] Marti-Bonmati L., Blanquer I., Tsiknakis M., Tsakou G., Martinez R., Capella-Gutierrez S., Zullino S., Meszaros J., Bron E.E., Gelpi J.L. (2025). Empowering cancer research in Europe: The EUCAIM cancer imaging infrastructure. Insights Imaging.

[6] Kitaguchi D., Kosugi N., Ishikawa Y., Narihiro S., Enomoto T., Oda T., Takeshita N., Ito M. (2025). A multicentre randomized controlled trial exploring the clinical usefulness of an artificial intelligence-based anatomical navigation system. Br. J. Surg..

[7] Tejedor P., Denost Q. (2024). The integration of artificial intelligence with robotic instruments in surgical practice. Br. J. Surg..

[8] Gumbs A.A., Frigerio I., Spolverato G., Croner R., Illanes A., Chouillard E., Elyan E. (2021). Artificial intelligence surgery: How do we get to autonomous actions in surgery?. Sensors.

[9] Gumbs A.A., Grasso S.V., Chouillard E., Croner R., Dagher I., Spolverato G., Frigerio I., Milone L., Khalpey Z., Rashidian N. (2025). Announcement of consensus conference on definitions of artificial intelligence for the next generation of surgeons. Artif. Intell. Surg..

[10] Ozmen M.M., Oviedo R.J. (2026). Wisdom, balance, and action: An ancient Anatolian perspective on leadership in AI-enabled surgery-a narrative mini-review. J. Robot. Surg..

[11] Rashidian N., Abu Hilal M., Frigerio I., Guerra M., Sterckx S., Tozzi F., Capelli G., Verdi D., Spolverato G., Gulla A. (2025). Ethics and trustworthiness of artificial intelligence in hepato-pancreato-biliary surgery: A snapshot of insights from the E-AHPBA survey. HPB.

[12] Kewalramani D., Loftus T.J., Mayol J., Narayan M. (2024). Artificial intelligence in surgery: A global balancing act. Br. J. Surg..

